# Odonata: Who They Are and What They Have Done for Us Lately: Classification and Ecosystem Services of Dragonflies

**DOI:** 10.3390/insects10030062

**Published:** 2019-02-28

**Authors:** Michael L. May

**Affiliations:** Department of Entomology, Rutgers University, New Brunswick, NJ 08901, USA; may@aesop.rutgers.edu

**Keywords:** damselfly, dragonfly, biomimetic technology, climate warming, ecological indicators, mosquito control, myth and art, phylogeny, predation

## Abstract

Odonata (dragonflies and damselflies) are well-known but often poorly understood insects. Their phylogeny and classification have proved difficult to understand but, through use of modern morphological and molecular techniques, is becoming better understood and is discussed here. Although not considered to be of high economic importance, they do provide esthetic/spiritual benefits to humans, and may have some impact as predators of disease vectors and agricultural pests. In addition, their larvae are very important as intermediate or top predators in many aquatic ecosystems. More recently, they have been the objects of study that have yielded new information on the mechanics and control of insect flight.

## 1. Introduction

Dragonflies are insects that people notice. As adults they are large, diurnal, often colorful, and are swift, acrobatic fliers. They are sufficiently noticeable that they have received numerous folk names, for example, in North America, mosquito hawk, horse stinger, snake doctor, adderbolt, darning needle, among many others [1]. They have become embedded in folklore and mythology in many cultures (see below) and are the subjects of beautiful art and depressingly tacky shlock (whoever started the idea that odonates have curly antennae?!).

## 2. Classification

Despite being so well known as a group, distinctions among species and even higher taxa often seem to be overlooked by the public. More than once, while I was collecting or observing Odonata in the field, I have encountered interested non-entomologists. Most people were bemused (rarely alarmed) by what I was doing and not infrequently asked about dragonflies. Many asked specifically about how many species occur in New Jersey (where I did most of my research). When I would say about 180 species, they were invariably flabbergasted—the usual first guess was about five or 10 species. It is a stark example of the fact that most of us see only a narrow slice of reality.

In fact, Odonata is a small order, by insect standards, with roughly 6300 species worldwide [2], allocated among three suborders: Zygoptera (damselflies, ca. 3200 spp.), Epiophlebioptera (3 spp.; perhaps part of the otherwise extinct suborder Anisozygoptera), and Anisoptera (ca. 3100 spp.) [2]. The Odonata plus Ephemeroptera have traditionally been placed together in Palaeoptera (insects that cannot fold their wings horizontally over their back). Alternatively, Odonata plus Neoptera have been considered to be a monophyletic taxon (= Metapterygota) that is sister to Ephemeroptera, or Ephemeroptera plus Pterygota (= Opisthoptera or Chiastomyaria) placed as sister to Odonata (reviewed in [3]). The most recent and extensive molecular phylogenies of Insecta as a whole [4,5] suggests that Palaeoptera and Neoptera are, after all, monophyletic sister taxa.

Linnaeus [6] placed all Odonata (he recognized 18 species) in his *Classis Insecta*, *Ordo Neuroptera*, and in a single genus, *Libellula*. Fabricius [7] was first to establish formal subdivisions within Odonata, erecting the new genera *Aeshna* (including species of our present-day aeshnids and gomphids) and *Agrion* for *Libellula virgo* L. and *Libellula puella* L., and in 1793 established what we now consider an order, the Odonata [8]. Leach [9] was the next to advance classification markedly by establishing families similar to those today, here listed with the included genera: (1) In Libellulida—*Libellula* L., *Cordulia* gen nov.; Aeshnides—*Gomphus* gen. nov., *Cordulegaster* gen. nov., *Aeshna* Fabr., *Anax* gen. nov.; (2) in Agrionida—*Agrion* L., *Lestes* gen. nov., *Calepteryx* gen. nov. (later amended to *Calopteryx*). Thus, the type genera of families occurring in Europe and North America (plus *Anax*, which has remained in Aeshnidae) were all described, although the families were not all recognized as such.

Montgomery [10] suggested, with good reason, that the years 1839–1842 saw the establishment of Odonata as a distinct subject of study in their own right. In 1839, Burmeister [11] described 159 spp. in six genera and Say [12] published the first work on North American “Neuroptera” (including Odonata). Rambur [13] described some 360 species in 33 genera, almost certainly the greatest number described in a single work. Perhaps most important, in 1840, the Belgian aristocrat, Baron Edmond de Sélys Longchamps, published, in collaboration with Hermann Hagen, Monographie des Libellulidées d’Europe [14], the first publication that attempted to treat all the Odonata of Europe, including previously and newly described species. Over the next 60 years, Selys published over 120 works on Odonata, the last [15] published posthumously. During this long period, he described 707 new species and named many new families and subfamilies, especially in what is now Calopterygoidea. With Hagen, he wrote monographic treatments of the known Calopterygidae [16] and Gomphidae [17] and on his own some 26 so-called “synopses” of all the then-recognized families, except Libellulidae. Most of the family-group taxa that were generally accepted throughout much of the 19th and nearly all of the 20th century were recognized or erected by Selys, although not always at the same rank. Selys also emphasized, more strongly than his predecessors, characteristics of wing venation as revealing taxonomic relationships [10], an emphasis that has subsequently been very valuable and yet in some cases seriously misleading [18]. At the time of his death, many species in his collection had still not been described. This was partly rectified by the late appearance of commissioned works by Ris [19] and Martin [20,21] that remain valuable today.

The work of Selys and his predecessors and contemporaries resulted in classification schemes that implied relationships among taxa, but even after Darwin, little attention was given making these explicit. Finally, in 1903 Needham [22] attempted a “geneologic” study of Odonata, especially Anisoptera. His eventual scheme was very like that implied by Selys, with the Anisoptera comprising two families, Libellulidae, including subfamilies Macromiinae, Corduliinae, and Libellulinae, and Aeschnidae [sic], including Gomphinae, Cordulegasterinae, Chlorogomphinae, Aeschninae, and several extinct taxa. His Zygoptera encompassed Calopterygidae and Coenagrionidae (the name *Coenagrion* was introduced by Kirby [23] owing to disputed priority of *Agrion*); Coenagrionidae included Lestinae and Coenagrioninae, and was approximately equivalent to Selys’ Agrioninae. In arriving at these conclusions, he relied entirely on characteristics of wing venation, which he interpreted primarily based on patterns of tracheation in the corresponding nymphal wing (a method that has subsequently been largely abandoned as unreliable). He rarely considered fossils, which he found too incomplete and hard to interpret. Later his student, Munz [24] made a similar but more detailed analysis of Zygoptera, with very similar conclusions.

Toward the end of the 19th and during the early 20th century a new source of evidence about Odonata and their evolutionary progenitors became available with the discovery of remarkable fossils, the remains of enormous, dragonfly-like insects, especially from the coal measures of France [25] and rich Permian deposits in the United States, from Kansas and Oklahoma [26]; Beckemeyer [27] gives a concise history of the latter discoveries. A few fossil Odonata were known earlier in the 19th century (e.g., [28]), but the size, age, degree of preservation, and evident similarities with modern Odonata made the new finds particularly intriguing. These so-called “giant dragonflies” are not, in fact, regarded as belonging to Odonata, because their wings have a very weak oblique nodus and lack a pterostigma and discal cell(s), but are usually placed in a separate order that is sister (or possibly ancestral) to Odonata. They are often called the Protodonata, but the correct name, owing to a quirk of priority, is Meganisoptera Martynov, 1932 [29].

With the discovery of numerous fossils, efforts to understand their place within Odonata or its progenitors and to understand the implications for classification of modern odonates were accelerated, Needham’s reservations notwithstanding. Among the major figures in this enterprise were R. J. Tillyard [30,31,32] and F. C. Fraser [31]. Both relied almost entirely on wing venation, as did many others (but see Kennedy [33,34]), since most fossils consisted only of wing impressions. This culminated in Fraser’s [35] revision of the order, which was influential for many years. He argued that Zygoptera are paraphyletic relative to Anisozygoptera and Anisoptera and apparently regarded Anisozygoptera as the direct ancestor of Anisoptera. Of course, Fraser did not use the term “paraphyletic” and was evidently unaware of Hennig’s early work (e.g., [36]). His conclusions were based largely on his earlier work with Tillyard and in particular on the characteristics of the Permian fossil wings found by Tilllyard [37,38]; these appeared very much like those of modern Zygoptera, with a pterostigma but without a well-developed nodus or closed discal cells. 

Fraser [35] produced one of the first phylogenetic trees (i.e., a branching diagram showing hypothesized relationships of ancestry and descent) ever applied to Odonata. By current standards it is an unusual tree with, for example, a paraphyletic Zygoptera; several extant taxa shown as ancestral to other extant taxa without branching, and, as noted, several stems that produce multiple branches (e.g., Lestoidea—his “Lestinoidea”—are immediate ancestors of the Calopterygoidea plus all the Anisoptera, whereas Coenagrionoidea arise independently from Protozygoptera, although the latter are probably not monophyletic [39]). The principal flaw; however, is that, as Fraser said explicitly in a slightly earlier short paper [40], his conclusions were based on “evidence of persistent archaic characters” rather than what we would now call synapomorphies. In common with many systematists of that time, he sought characters in extant species that were shared with their presumed ancestors. Nevertheless, his conclusions were widely accepted, although in much of North America Needham’s scheme predominated, probably in part because of the publication of [41].

Odonata classification thereafter changed little for more than 30 years, despite the publication in English of Hennig’s major work [42] in 1966. Several other hypotheses were proposed during the 1980’s–2000’s; however, to describe the phylogeny of all or part of the Odonata. In 1991, Pfau [43] proposed a revision of the Anisoptera based on his scenario for the evolution of the male secondary genitalia, especially the genital ligula; it was dependent on the judgement that physical forces involved in the movement of the penis and ejection of sperm dictated a one-way series of morphological changes that could not be reversed without negating their function. He later elaborated this classification further [44] (see his Figure 65). While his morphological analysis is unquestionably a *tour de force*, his phylogenetic conclusions are contradicted by other studies using a variety of characters, including DNA sequence data (see below), and have not been generally accepted. 

In 2003 Rehn [45] consistently recovered both Anisoptera and Zygoptera as monophyletic, using a wide selection of both Anisoptera and Zygoptera, including a number of fossil taxa. His analysis was based on morphological characters, parsimony as the criterion for acceptance, and two alternative character weighting schemes. Anisopteran relationships were nearly constant in all analyses, although in Zygoptera relationships were unstable within the Calopterygoidea (presaging things to come). Lestoidea and Platystictidae were sister to Coenagrionoidea. Importantly, Zygoptera was always sister to Anisoptera plus *Epiophlebia*. Rehn also reviewed three earlier morphology-based studies [46,47,48] and included abbreviated versions of their trees for comparisons with his results.

In 1986, Carle [49], using morphological characters, revised the Gomphidae. Characters were not illustrated, and Carle employed an unusual tabular method of recognizing and evaluating synapomorphies; others have generally found this difficult to use, and it has not been widely tested. Nevertheless, a more recent partial revision of the family based on molecular data and more standard methods of analysis [50] has borne out many of his conclusions.

Carle and Louton’s [51] discovery of the larva of *Neopetalia punctata* showed clearly that that species is related to *Cordulegaster* and is not an aeshnoid, as previously thought. They then established a new family, Austropetaliidae, to accommodate species from Australia and southern South America that actually are close relatives but are unrelated to *Neopetalia*, with which they had been placed. Subsequently Carle [52] revised the “ancient Gondwanian libelluloides”, using techniques similar to [49]; parts of this work remain controversial. Von Ellenrieder [53] published a nearly comprehensive and very well illustrated phylogeny of Aeshnidae based on parsimony criteria and morphological characters. This is much more complete than any previous or subsequent analysis of the family. Finally, Lohman [54] revised Cordulegastridae, also using parsimony and morphology; this was rather sparsely illustrated and established a number of new subsidiary taxa whose validity has been questioned. It certainly needs and deserves independent confirmation.

With continual advances in computer-assisted tree building and automated DNA sequencing, phylogenetic analysis has become faster, computationally more accurate, and somewhat less dependent on ad hoc assumptions about character inclusion and weighting. Nevertheless, many analyses have been mutually incongruent, and, in aggregate, failed to resolve some important issues. Trueman [55] summarized many of these studies and showed the resultant trees, redrawn and at the family level—a sobering exercise owing to their frequent discordance. As he pointed out, one general problem of morphology-based trees is that venational patterns are very difficult to homologize on purely structural grounds, so that homology assessments often depend on a pre-supposed phylogeny. Even molecular-based trees were incongruent among themselves, however, and Misof et al. [56] showed that tree topology could depend on outgroup choice and among-site homogeneity in substitution rates.

Over the last decade, however, considerable progress has been made in further understanding odonate phylogeny and thus constructing a more-or-less “natural” classification. I can only describe a few of the more comprehensive of these studies, but these provide a fairly good picture of where we stand currently. The first of these [57], by Ware et al., was the most tightly focused, including only the Libelluloidea, rooted by Neopetaliidae, Cordulegastridae, and Chlorogomphidae (which one could justify calling plesiotypic libelluloids). Beyond that point, a clade was identified that had not been recognized as a taxon in earlier work (or rather was seen as one family, Synthemistidae, and three erstwhile subfamilies of Corduliidae: Cordulephyinae, Idionychinae, and Gomphomacromiinae) [35]. Ware et al. designated this as the GSI clade (for *Gomphomacromia*, *Synthemis*, and *Idionyx*), since they were not then confident of its composition, although support for that branch of the tree was quite high. At the next split, Corduliidae was found to be sister to Macromiidae plus Libellulidae. *Hemicordulia* and *Procordulia*, sometimes placed in a separate family, were nested well within Corduliidae. Macromiidae was usually regarded as a subfamily of Corduliidae until Gloyd [58] elevated it, and it is still regarded as a subfamily by some. Here it is clearly distinct from Corduliidae and actually appears to be closer to Libellulidae, although support for this position is moderate at best.

Bybee et al. [59] included all major groups of Odonata, fossil as well as extant taxa, in their phylogeny, thus by necessity using both morphological and molecular data. They found Zygoptera and Anisoptera to be monophyletic, with *Epiophlebia* as the extant sister group to Anisoptera. Relationships within Anisoptera were closely similar to those described in [57]. Lestoidea was recovered as sister to all other Zygoptera, with all its constituent families (Lestidae, Synlestidae, and Austrolestidae) monophyletic except that Perilestidae was not monophyletic in their parsimony tree. Beyond Lestoidea, the next branch led to Platycnemididae, which was unexpected, since the latter was previously thought to be related to Protoneuridae. Beyond that point, three successive major branches led to Chlorocyphidae, *Devadatta*, and Calopterygidae, respectively. The terminal portions of the phylogeny included one branch with a then somewhat unexpected mixture of calopterygoid, amphypterigid, and megapodagrionid genera, which was sister to a branch including the Isostictidae (a family with extremely reduced venation), Platycnemididae, Coenagrionidae, Pseudostigmatidae, and Protoneuridae. Additionally noteworthy are the facts that Protoneuridae appeared at two places, one as sister to Platystictidae and one within Coenagrionidae, and Pseudostigmatidae (the “giant” or “helicopter” damselflies of the Neotropics) was also nested within Coenagrionidae.

In the most recent extensive revisions of Odonata, Zygoptera and Anisoptera have been treated separately, since a substantial body of evidence now indicates that they are each monophyletic, and it appears very likely that Zygoptera is sister to *Epiophlebia* plus Anisoptera. Supplementary Figure S1 is taken from [60] and summarizes our best current understanding of zygopteran phylogeny (Dijkstra et al. initially produced a species-level tree, too large to be reproduced here, then reduced it to family-level if existing families were recovered). The genera, labeled *incertae sedis*, did not fit clearly within a known family in their analysis. The implied classification, as part of a proposed classification for all Odonata, was also published [61].

Important features of this phylogeny are that Lestoidea (including the very plesiotypic *Hemiphlebia* [35,48]) is sister to all other Zygoptera, and Platystictidae, now placed in its own superfamily, is sister to all the remaining non-lestoid Zygoptera. Thereafter the Calopterygidae hit the fan: Calopterygoidea, Amphipterygidae, and Megapodagionidae are split into 19 small families and eight unassigned genera, producing a large polytomy comprising about 800 species. Finally, the terminal taxa are Isostictidae and Coenagrionoidea (the latter includes Platycnemididae plus Coenagrionidae). Notably, as in [59], “Protoneuridae” were divided into a new-world clade (including *Protoneura*) nested within Coenagrionidae and an old-world clade within the Platycnemididae; these were named Protoneurinae and Disparoneurinae, respectively. Additionally, the supposed subfamily “Argiinae” was split into a new-world (*Argia*, in Coenagrionidae) and two old-world components, with the latter split between two subfamilies in Platycnemididae, Onychargiinae and Idiocnemidinae. “Pseudostigmatidae” is nested within Coenagrionidae, also as in [59]. Several of these changes had also been anticipated in [62]. Understanding the remaining uncertainties in Zygoptera classification is an ongoing problem. 

Happily, Anisoptera appear to be somewhat less confusing. Carle et al. [63] analyzed the suborder (results are summarized in Supplementary Figure S2) and at the family level verified many of the results already described [35,58]. Using a taxonomically-diverse selection of Zygoptera as the outgroup, *Epiophlebia* was sister to Anisoptera and Aeshnoidea (Austropetaliidae plus Aeshnidae) was found to be sister to all other Anisoptera. Despite the much smaller taxon sample, the aeshnid phylogeny was not materially different from that in [53].

Among non-aeshnoids, Petaluridae is most likely (Bayesian posterior probability = 74%) sister to Gomphidae but, because of a relatively long branch leading to Gomphidae and because all the remaining 26% of the Bayesian trees recovered Petaluridae as sister to Cordulegastroidea, the position of petalurids is somewhat uncertain, as reflected in Figure S2. The Gomphidae are under sampled, but the principal groups are similar to those of Carle [48], except that his subfamily Octogomphinae was not recovered. This section of the phylogeny in [63] is also very similar to that in [49].

The cordulegastroids formed a pectinate array that was sister to Libelluloidea. Morphological evidence suggests that these families might, in fact, be paraphyletic relative to Libelluloidea [50], so this relationship also requires some additional examination.

As expected, Libelluloidea *s.s*. form the terminal portion of the tree. Within this very large taxon, the overall pattern is quite similar to that found by Ware et al. [57]. In particular, it recovered the “GSI” clade, containing gomphomacromiines, synthemistines, and representatives of several other ostensible subfamilies of Corduliidae established by Tillyard and/or Fraser, including Idomacromiinae, Idionychinae, and Cordulephyinae. The morphology of the included species is rather diverse, and this taxon deserves yet more extensive examination, although it has moderate to very high support in both [57] and [63]. Macromiidae and Corduliidae both occupy traditional positions relative to Libellulidae. *Pentathemis* and *Aeschnosoma* are grouped together and are relatively distant from other Corduliidae, as suggested in [64].

Libellulidae is the most intensively studied family, but classification within the family is still unsettled, in part because of morphological similarities, in a few cases involving rather strong apparent synapomorphies, among seemingly divergent subfamilies (e.g., *Zenithoptera, Palpopleura,* and *Diastatops*), strong morphological divergence among species assigned to the same subfamily (e.g., *Pantala* and *Trithemis*), and simply the large number of species, so thorough taxon sampling is difficult. Nevertheless, tentative subfamily designations have been assigned [62]. These are fairly concordant with some other results based on molecular genetic data [57,65,66] in assignment of genera, but not necessarily in branching order within the family. All need further investigation, but it seems very likely that earlier attempts to classify libellulids based on morphology alone require extensive revision.

Now, having finally disposed of the dramatis personae, we should proceed to at least a small part of the drama itself.

## 3. Ecosystem Services

**Figure 1:**
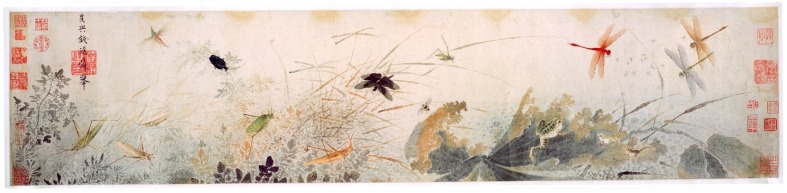
Early Autumn, painting by Qian Xuan (China, 1235–1305, Yuan Dynasty; [69]. Reproduced from https://en.wikipedia.org/wiki/Qian_Xuan, visited on 2 November 2018).

The simplest and most straightforward definition of ecosystem services is that given in the Millennium Ecosystem Assessment [67]—“the benefits provided [to humans] by ecosystems”. To my mind, the greatest benefit of Odonata is simply that they exist. They have, in fact, inspired visual art and literature, especially in Asia, for hundreds of years, as seen in the painting reproduced below (Figure 1) and in poetry such as the haiku by Issa (Japan, 1763–1828; translation by S. King in [68]).

From my shoulder

To the Buddha’s shoulder—

The dragonfly

Dragonflies figure quite commonly in haiku and are important in Japanese arts and culture generally (e.g., [70]; the internet has a great deal of information on this subject, including the very entertaining and informative [71] “Vegder’s Blog”, https://printsofjapan.wordpress.com/category/japanese-painting (visited on 2 November 2018)—look down the sidebar under Top Posts for “The Dragonfly”; see also Cultural Odonatology [72]. www.ucsd.edu/archive/personal/ron/CVNC/odonata/cultural-odonatology.html, visited on 2 November 2018).

**Figure 2:**
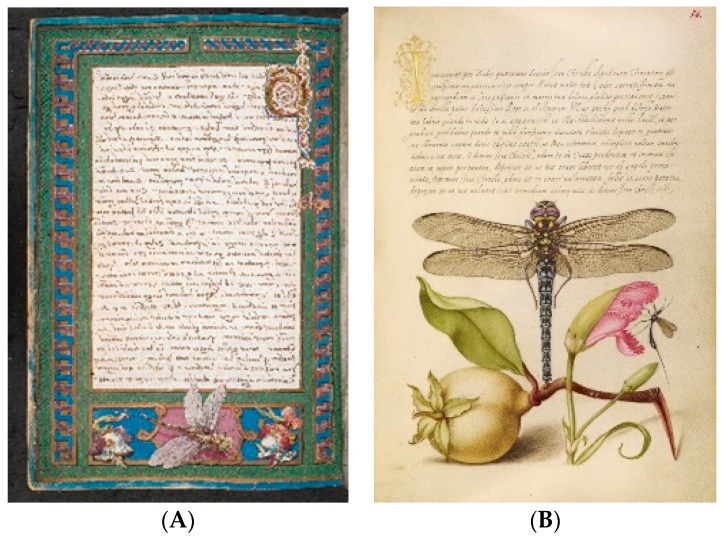
(**A**) Page from Caesar’s *De bello Gallico* with added border incorporating a dragonfly and emblems of the Visconti family [74]; (**B**) Dragonfly, Pear, Carnation, and Insect in *Mira Calligraphiae Monumenta*, 1561–1562, illumination added 1591–1596 by Joris Hoefnagel; J. Paul Getty Museum, Ms. 20, fol. 76 [75].

Odonata are much less prominent in Western literature and art but do appear, for example, in Tennyson’s well-known poem, *The Dragonfly*, among others. In recent years they have become popular as motifs in popular culture. Interestingly, they were also surprisingly frequent in medieval and renaissance illuminated manuscripts (Figure 2, and see [73]).

Dragonflies have also been important in the culture of some Native American groups. Perhaps the Zuni tale of the creation of dragonflies is best known because of Tony Hillerman’s children’s book, *The Boy Who Made Dragonfly* [76]. In the Southwest, native people valued dragonflies as signs of water, fertility, and abundance, and they were frequent motifs on Mimbres pottery [77]. On the northern Plains, the Cheyenne and Dakota viewed dragonflies as good omens and protection against death, and they were often represented on clothing and on dwellings and war shields [78,79].

Appreciation of Odonata, both aesthetic and scientific, seems to have undergone a dramatic expansion over the last two or three decades. Several international organizations that promote interest in and study of dragonflies now exist (e.g., [80]; which also lists many resources, including contact information for other societies and publications). The internet, digital photography, and the development of a number of excellent field guides (e.g., [81,82,83]) have undoubtedly given impetus to this trend. The examples of field guides given are only a few of many in English, and high-quality guides exist also in a number of other languages. It is probably fair to say that both professional and amateur engagement with Odonata is at an all-time high. It is particularly encouraging that interest seems to be expanding rapidly in hitherto understudied regions, such as South and Central America (e.g., Sociedad de Odonatología Latinoamericana).

Simaika and Samways [84] were probably the first to discuss odonates explicitly within the framework of ecosystem services. They, too, recognized the value of what they called “Cultural Services”, which include art and symbolic meaning. In addition, they note that in recent years, interest in nature in general and Odonata in particular has led to the publication of many regional field guides enabling enthusiasts to identify species in the field, as well as the establishment of dragonfly reserves in Japan and of viewing areas and dragonfly trails elsewhere [85]. Furthermore, dragonflies are measurably attractive to visitors at recreation sites, and their presence can overcome some negative impacts such as excessive debris in such areas [86]. Lemelin [87] reviewed many other instances of recreational and outdoor experiences enhanced by the presence of odonates.

Dragonflies do also provide more material services and, rarely, disservices. The first of these to come quickly to mind is probably, “Oh, they eat mosquitos” (see Figure 1). This is certainly true, and is undoubtedly beneficial since mosquitos are at best annoying and at worst dangerous vectors of serious and widespread diseases. One of the earliest attempts to determine the potential for odonates to reduce mosquito and house fly populations resulted in the publication of *Dragon Flies vs Mosquitoes: Can the Mosquito Pest Be Mitigated?* [88]. The text consists of several separate accounts by entomologists and others, most of whom concluded that attempts at control using dragonflies would be futile. Nevertheless, the idea persisted that dragonflies should be effective in suppressing mosquitos. It is clear that dragonflies can detect and will exploit dense swarms of Diptera, including mosquitos and stable flies [89,90], and they capture prey in a remarkably high percentage of attempts [91,92]. There is; however, substantial doubt that dragonflies materially reduce populations of mosquitoes or other pests, except perhaps very locally. Although both adults and larvae are highly predaceous, both stages are generalists and, to a considerable degree, tend to feed on the most abundant available prey. Blois [93] found that three species of European Anisoptera larvae fed selectively on certain prey taxa and avoided others but always took several types of prey, and their preference varied with species, instar, and time of year (Blois did not provide mosquito larvae). In addition, the larvae are often highly cannibalistic, which may restrict their natural population density. In addition, the life cycles of Odonata are commonly considerably longer than those of mosquitoes and other dipteran pests, potentially slowing their population response to pest outbreaks.

Several studies, mostly in the laboratory (e.g., [94,95,96,97,98]) but also in mesocosms [99,100] and irrigation ditches [101], have suggested that odonate larvae might play a role in control of other insects that also have an aquatic larval stage, including mosquitoes. Most experiments in an outdoor setting involved introducing new or augmenting existing population of Odonata. Younes, et al. [102], also based on laboratory experiments, concluded that *Hemianax ephippiger* larvae might successfully control populations of snails that are the secondary host of the human parasite, *Schistosoma*. Stav et al. [103], using small mesocosms (plastic tanks) that mimicked natural rock pools where the mosquitoes breed, found that *Culiseta longiareolata* females oviposited less frequently in tanks containing an unconfined *Anax imperator* larva and that mosquito larvae that did hatch were mostly consumed by the *Anax* larvae. The effect of *A. imperator* on widespread *Culiseta* populations is not known. Some fairly strong evidence suggests that multiple predators, including Odonata larvae, can depress prey populations significantly more that equivalent numbers of a single predator, as shown in mesocosm experiments [104] and inferred to be true of predators, including dragonflies, of culicid larvae in natural saline pools in Western Australia [105].

The only study that has definitively shown that Odonata can control or even eliminate pest populations is that of Sebastian et al. [106], in which larvae of *Crocothemis servilia* were introduced into household water containers in a carefully surveyed neighborhood in Yangoon, Myanmar, in an attempt to suppress a local population of *Aedes aegypti*; another neighborhood was monitored but not treated. Residents of the treatment neighborhood were educated about the aims and techniques of the experiment, and they supported the project. *Crocothemis* larvae were obtained by rearing eggs of wild-caught females to an age of four weeks, then transferring four larvae into each potential breeding container. Within about two months of the introduction of the dragonfly larvae, the density of *Aedes* larvae had declined to less than 5% of its initial density and the density of adult females resting in dwellings was only about 15–20% of that in untreated sites. Thus, suppression of mosquitoes was very successful but depended on a program of augmentative release that was labor intensive, and also depended on wholehearted cooperation from the human population. At present the only clear evidence that unmanipulated Odonata populations regularly suppress prey populations is the reduction of treehole mosquito larvae by cohabiting pseudostigmatine damselfly larvae [107].

Laboratory observations or experiments with adult Odonata are rarely possible, but information on the diet inferred from gut contents of wild-caught adults shows that, although small Diptera are an important part of their diet, mosquitoes are seldom taken in numbers that suggest dragonfly predation might diminish their populations [108,109].

Of course, Odonata feed on many other insects, undoubtedly including other disease vectors and some crop pests. For instance, adult *Anax junius* have been recorded consuming large numbers of stable flies [85], and Neal and Whitcomb [110] recorded many adult dragonflies in Florida soybean fields, with *Erythemis simplicicollis* especially taking *Heliothis zea* and other noctuid moths. Odonata are also abundant at certain times of year in rice paddies in Japan and Southeast Asia and may feed extensively on rice pests [111,112,113] (all cited in [114]). While odonates are not, in general, known to reduce populations of crop pests, Yasumatsu et al. [113] observed that pesticide use is much reduced in paddies with abundant dragonflies. It may be that they, perhaps in concert with other predators, may truncate population peaks of herbivorous insects [114]. Che Salmah et al. [115] suggested that the common libellulid, *Neurothemis tullia*, might be “an effective predator in rice fields”; however, they did not demonstrate an effect on pest populations.

The feeding habits of Anisoptera do occasionally have negative effects from a human perspective. Large aeshnids have frequently been found to feed on honeybees, sometimes patrolling near a hive and picking off workers as they emerge to forage and seriously curtailing the number of foragers [116]. They also may do harm by consuming wild pollinators. Knight et al. [117] studied eight ponds in northern Florida, four with fish and four fish-free. They discovered that flowers near the ponds with fish received many more pollinator visits and were less subject to pollen limitation than the same species near fish-free ponds. Dragonfly larvae, and hence adult dragonflies, were much more abundant in or near the fish-free ponds and, because they preyed heavily on pollinators, they significantly reduced pollination of insect-pollinated plants near their natal ponds. Thus, dragonflies mediated the strong correlation between fish and pollination. How commonly this relationship occurs is unknown, but this was one of the earliest studies to clearly demonstrate a trophic cascade across ecosystem boundaries.

A near mirror image of the study just described is [118]. Stock tanks (1300 L) served as mesocosms, each stocked with equivalent numbers of macrophytes, snails, and zooplankton, and each surrounded by four small (100 L) pools in which purple loosetrife (*Lythrum salicaria*) were planted. The number of flowers surrounding each mesocosm was controlled at one of four levels for eight weeks, during which potential pollinators of *Lythrum*, adult dragonflies, and dragonfly oviposition events were assessed weekly and plankton and macroinvertebrates were sampled at the end of the study. The eventual results showed that pollinators, adult dragonflies, oviposition, and plankton species richness (but not plankton abundance) all increased with increasing flower availability. Thus, it appears that effects mediated by Odonata affect both terrestrial and aquatic organisms across ecosystems.

The possibility that animals with complex life cycles (i.e., with one or more life stages occurring in one ecosystem and other stages in a different ecosystem, as in [48,49]) has drawn a good deal of attention (e.g., [119,120,121]). Some effects of the emergence of dragonflies on terrestrial riparian systems has been examined in mesocosms with flow maintained by pumps [122]. *Pantala flavescens* very quickly naturally colonized the mesocosms. Other aquatic invertebrates and phytoplankton were introduced, and fish were placed in some mesocosms. When present, fish reduced emergence of *Pantala* (which generally do not coexist with fish) by about 50%, thus presumably reducing subsequent predation pressure on riparian aerial prey compared to that adjacent to fish-free mesocosms. Diptera avoided ovipositing in mesocosms containing fish alone, fish and dragonfly larvae, or dragonfly larvae alone, thus reducing local emergence of potential prey of terrestrial organisms [123]. This generalizes the finding [103] that *Culiseta* mosquitos avoid pools containing dragonfly larvae. Oviposition by Diptera was also reduced in mesocosms without dragonflies but closely adjacent to those with dragonflies. Finally, a survey of streams in North and South America found the predatory insects, preponderantly Odonata, made up an increasing fraction of emergent insects, up to 86% of biomass, as stream temperature increased [124]. Thus, dragonflies may be especially important as terrestrial predators near watercourses in the tropics.

Another way in which insects like dragonflies affect both aquatic and terrestrial ecosystems is by transport of materials and energy. Transport into water bodies normally predominates simply due to gravity. This may be augmented, although usually only slightly, by eggs laid in water by animals with terrestrial adults but aquatic immatures, such as amphibians and many insects. On the other hand, the emergence of insects with aquatic larvae and terrestrial adults may transport material in the opposite direction, sometimes in quantities that make up an important fraction of the nutrients and reduced carbon that is available to nearby terrestrial communities (e.g., [125,126]). Few studies have given estimates of transport specifically by Odonata, but Kautza and Sullivan [127] found that mature adult coenagrionid damselflies had derived about 40% of their energy content from aquatic sources (i.e., carried over from larval energy stores or obtained from aquatic prey). Popova et al. [128] estimated that total secondary productivity of Odonata alone, at their study site in southwestern Siberia, was approximately equal to that of terrestrial insects in the same region. Thus, odonates may sometimes be responsible for a significant portion of aquatic-to-terrestrial transport.

An aspect of materials and energy transport that has not really been investigated for odonates is that resulting from long distance migration. An estimated 3200 tons of biomass in the form of migrating insects pass above southern England each year [129]. Dragonflies were probably not included in this figure, and surely their numbers alone are much smaller (although migratory flights estimated at 1 million individuals have been seen [130]). Nevertheless, they are, on average, large insects, the majority of their biomass is concentrated in or near fresh water, and some species, notably *Anax junius* and *Pantala flavescens*, appear in unusual concentrations near the terminus of their movements, as seen, for example, in Veracruz, MX (J. Matthews, pers. comm., 2005; D. Paulson, pers. comm. 2006) or southern Florida [131]. The effects of these seasonal effluxes and influxes to the aquatic habitats they occupy have not been investigated, but given that they are top or nearly top predators, it may extend beyond the effects of mass and energy transfer alone (whether this is an ecosystem service as defined above is unknown, but at the very least it should pique human curiosity).

Maintenance of viable and functioning ecosystems certainly can be considered an ecosystem service, in that it helps to preserve other services. Large dragonfly larvae are top predators in many fish-free water bodies [132]; different species of the zygopteran genus *Enallagma* occupy fish-free and fish-containing lakes; lakes that shift from having to lacking fish during some years (“winterkill” lakes) are dominated by still a different species of *Enallagma*. The species that coexist with fish have different and appropriate predator avoidance behavior than those in “dragonfly” lakes [133]. Moreover, *Enallagma* from both fish and fishless lakes disperse very little, while those from winterkill lakes disperse considerably more widely [134]. Thus, the identity of the apex predator makes a crucial difference in the populations of prey organisms [135], probably including many taxa in addition to damselflies, and may affect later terrestrial behavior.

Odonata are often good indicators of the quality and integrity of aquatic habitats, sometimes including adjacent riparian or littoral areas. Although some authors have assessed Odonata as poor indicators of water quality (e.g., [136]), they have been used successfully in this role for almost four decades [137]. Many adult odonates are highly visible and often identifiable to species on the wing, thus greatly facilitating such studies, although with the caveat that the diversity of larvae in a polluted stream may be considerably less than that of adults. Schmidt [138] pointed out that the presence of a characteristic assemblage of species may be more indicative of habitat quality than is any single species, and assessment of such assemblages have been used to gauge recovery of degraded wetlands [139]. Clark and Samways [140] showed that surveys based on adult male Odonata were quite useful at identifying habitat characteristics that were important or essential for maintenance of many species and are likely to have implications for conservation of other aquatic macroinvertebrates. Samways and colleagues developed the “Dragonfly Biotic Index” [141,142], which often can be an excellent index of aquatic habitat quality. Wildermuth [143] and Harabiš and Dolný [144] showed, again by surveying adult dragonflies over a period of time, that restoration of human-damaged habitats need to allow for continuing natural disturbances in order to truly recover the natural of aquatic populations in the area.

Several investigators have used Odonata as models to make, and ultimately test, predictions about the effects of global warming on dragonfly range [145,146,147]. The last authors suggested that in South Africa a few species might enjoy range expansions as a result of climate warning but many were likely to persist but suffer range contraction, especially at high altitudes. The other studies [145,146] predicted serious range reduction and possible extirpation in the high-altitude species they studied. Confirmation (or otherwise) will, of course, require continued observation over years or decades. In addition, documentations and analyses of recent range changes by Odonata have been quite successful [148,149,150,151,152] and provide data that is highly relevant to understanding how ongoing climate change affects aquatic animals.

To turn briefly to a relatively new and truly remarkable service of Odonata, dragonflies have inspired, and in some cases become part of, designs for and experiments on unusual modes of flight and visual flight control. Anyone who has watched Anisoptera fly, much less tried to net them, has surely been impressed by their maneuverability, speed, and visual guidance control. In fact, studies of Odonata have taught us a great deal about so-called unsteady aerodynamics (i.e., generation of aerodynamic forces that cannot be duplicated by summing the forces that one would expect from a series of static wing forms held successively in the positions and angles that they occupy at different moments during a wingbeat). Sophisticated aerodynamic analysis has shown that all phases of their flight, especially rapid changes of direction or velocity, depend on deformations controlled by the interaction of flight muscles and the detailed structure of the wings, interaction of the fore- and hindwings, and development of strong vortices along the leading edge of the wings (recently reviewed in [153]. These studies, along with a better understanding of how vision guides flight maneuvers [154,155], have enhanced our understanding of both aerodynamic principles and analysis and the mechanisms of insect visual systems.

This understanding has given rise to spectacular technological endeavors, perhaps most ambitiously in the DragonflEye project, developed jointly by Draper Laboratory, Inc., of Cambridge, MA, and Howard Hughes Medical Institute (HHMI) at Janelia Farm, Ashburn, VA. This has resulted in the development of a miniature “backpack” that can be glued to the back of a medium sized dragonfly in such a way that the insect can still fly; it is self-navigating, uses miniature solar panels for power, sends steering signals to the dragonfly brain via “optrodes” (tiny implanted light emitters) that stimulate steering neurons that have been genetically modified to respond to light. To my knowledge, the results to date have not been formally published, but some details are available online at IEEE Spectrum [156].

So, we have taken the “services” of dragonflies from the sublime to the—astonishing. At least, I hope it is clear that Odonata have long had and continue to have a role in and influence on human welfare, as we undoubtedly have on theirs.

## Supplementary Materials

The following are available online at https://www.mdpi.com/2075-4450/10/3/62/s1, Figure S1: The most recent extensive phylogeny of suborder Zygoptera; Figure S2: The most recent extensive phylogeny of the suborder Anisoptera.
