# Supplementary material for: Odonata: Who They Are and What They Have Done for Us Lately: Classification and Ecosystem Services of Dragonflies
Source: Insects. 2019 Feb 28;10(3):62. doi: 10.3390/insects10030062 (PMC6468591; doi:10.3390/insects10030062)
Supplement: Supplementary file 1 [file insects-10-00062-s001.pdf]

insects

MDPI

Review

Odonata: Who They Are and What They Have Done for Us Lately: Classification and Ecosystem Services of Dragonflies

Michael L. May

Department of Entomology, Rutgers University, New Brunswick, NJ 08901, USA; may@aesop.rutgers.edu

Received: 26 January 2019; Accepted: 22 February 2019; Published: date

Supplementary Materials

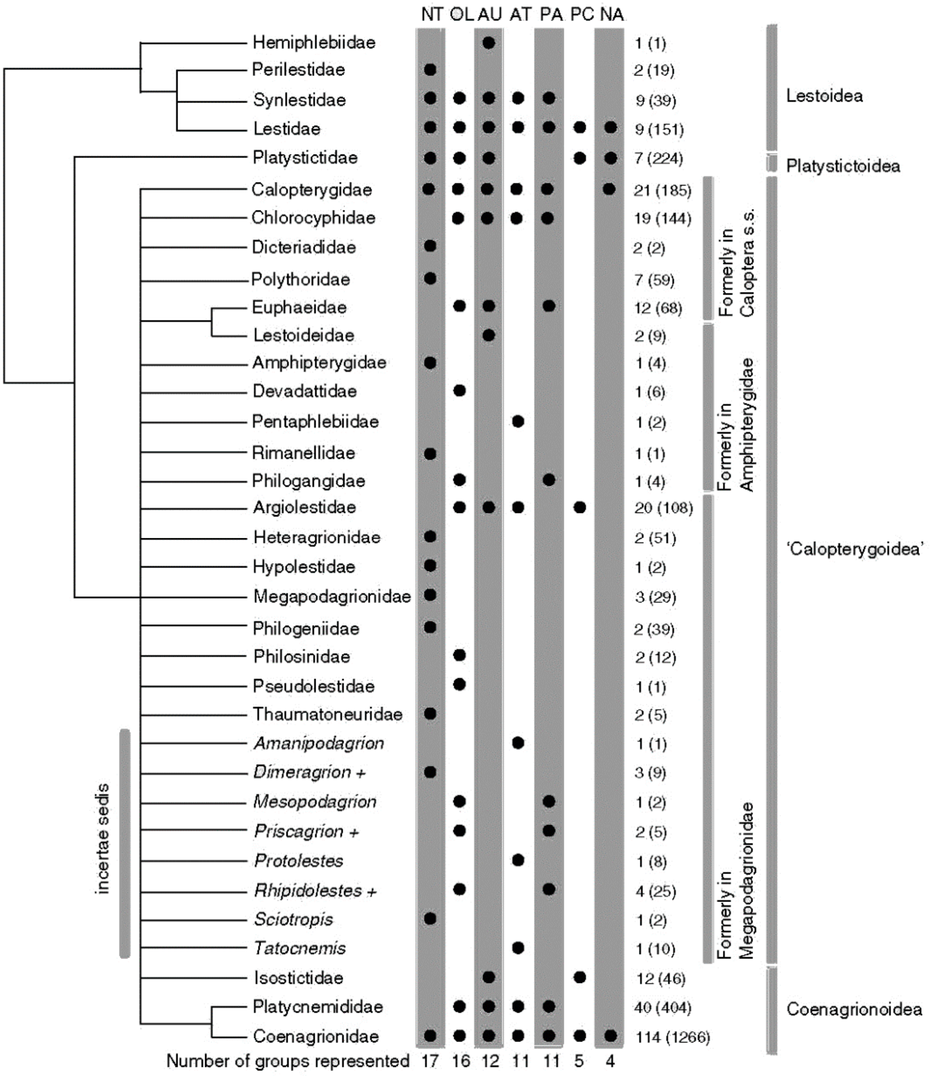

Figure S1. Abbreviated phylogram of Zygoptera. Only monophyletic family names are show; shaded area indicates geographic distribution. From Dijkstra, et al., 2013 [1].

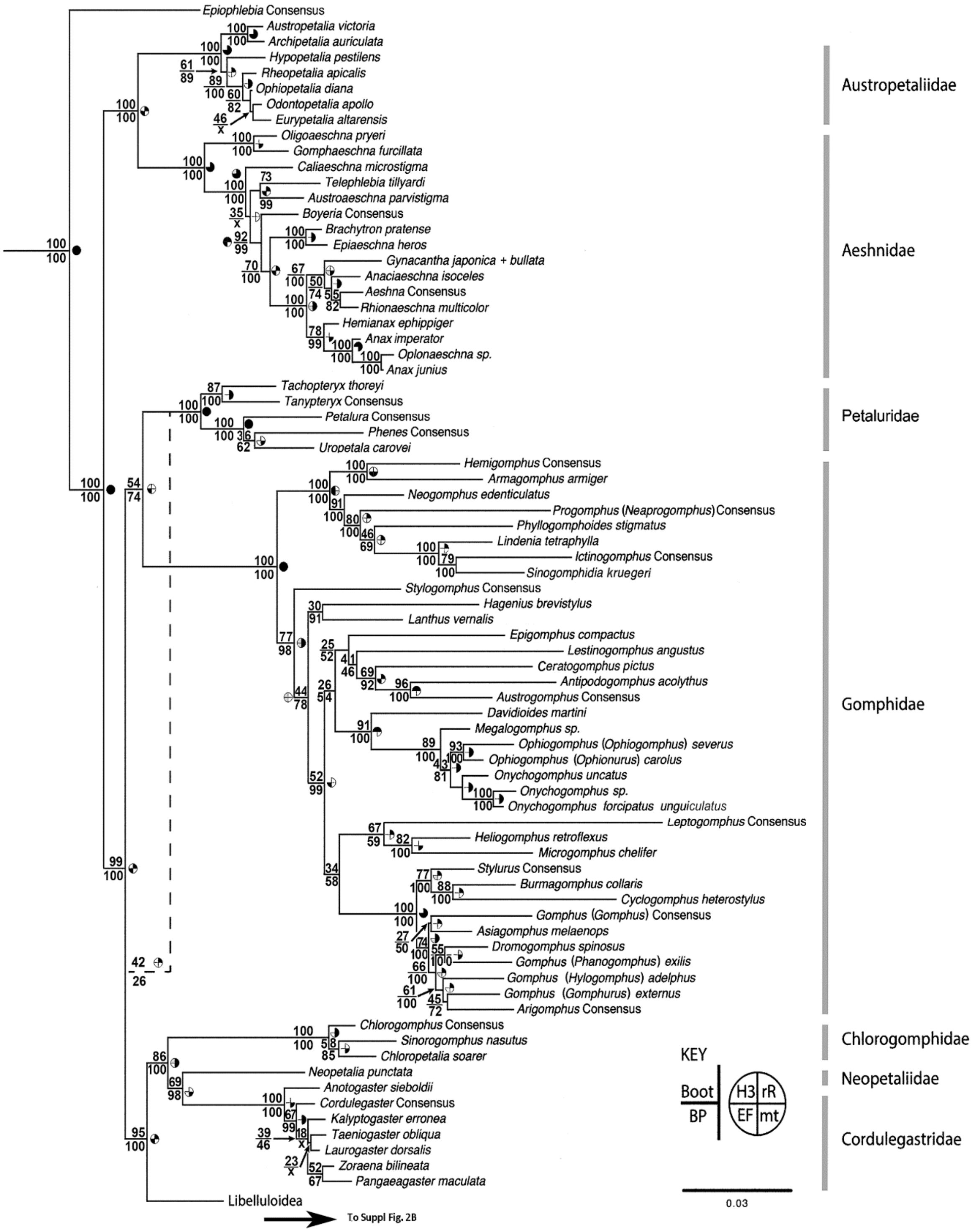
Epiophlebia Consensus
Austropetalia victoria
Archipetalia auriculata
Hypopetalia pestilens
Rheopetalia apicalis
Ophiopetalia diana
Odontopetalia apollo
Eurypetalia altarensis
Oligoaeschna pryeri
Gomphaeschna furcillata
Caliaeschna microstigma
Telephlebia tillyardi
Austroaeschna parvistigma
Boyeria Consensus
Brachytron pratense
Epiaeschna heros
Gynacantha japonica + bullata
Anaciaeschna isoceles
Aeshna Consensus
Rhionaeschna multicolor
Hemianax ephippiger
Anax imperator
Oplonaeschna sp.
Anax junius
Tachopteryx thoreyi
Tanypteryx Consensus
Petalura Consensus
Phenes Consensus
Uropetala carovei
Hemigomphus Consensus
Armagomphus armiger
Neogomphus edenticulatus
Progomphus (Neaprogomphus) Consensus
Phyllogomphoides stigmatus
Lindenia tetraphylla
Ictinogomphus Consensus
Sinogomphidia kruegeri
Stylogomphus Consensus
Hagenius brevistylus
Lanthus vernalis
Epigomphus compactus
Lestinogomphus angustus
Ceratogomphus pictus
Antipodogomphus acolythus
Austrogomphus Consensus
Davidioides martini
Megalogomphus sp.
Ophiogomphus (Ophiogomphus) severus
Ophiogomphus (Ophionurus) carolus
Onychogomphus uncatus
Onychogomphus sp.
Onychogomphus forcipatus unguiculatus
Leptogomphus Consensus
Heliogomphus retroflexus
Microgomphus chelifer
Stylurus Consensus
Burmagomphus collaris
Cyclogomphus heterostylus
Gomphus (Gomphus) Consensus
Asiagomphus melaenops
Dromogomphus spinosus
Gomphus (Phanogomphus) exilis
Gomphus (Hylogomphus) adelphus
Gomphus (Gomphurus) externus
Arigomphus Consensus
Chlorogomphus Consensus
Sinorogomphus nasutus
Chloropetalia soarer
Neopetalia punctata
Anotogaster sieboldii
Cordulegaster Consensus
Kalyptogaster erronea
Taeniogaster obliqua
Laurogaster dorsalis
Zoraena bilineata
Pangaeagaster maculata
Libelluloidea
To Suppl Fig. 2B
Austropetaliidae
Aeshnidae
Petaluridae
Gomphidae
Chlorogomphidae
Neopetaliidae
Cordulegastridae
KEY
Boot
BP
H3
rR
EF
mt
0.03

A

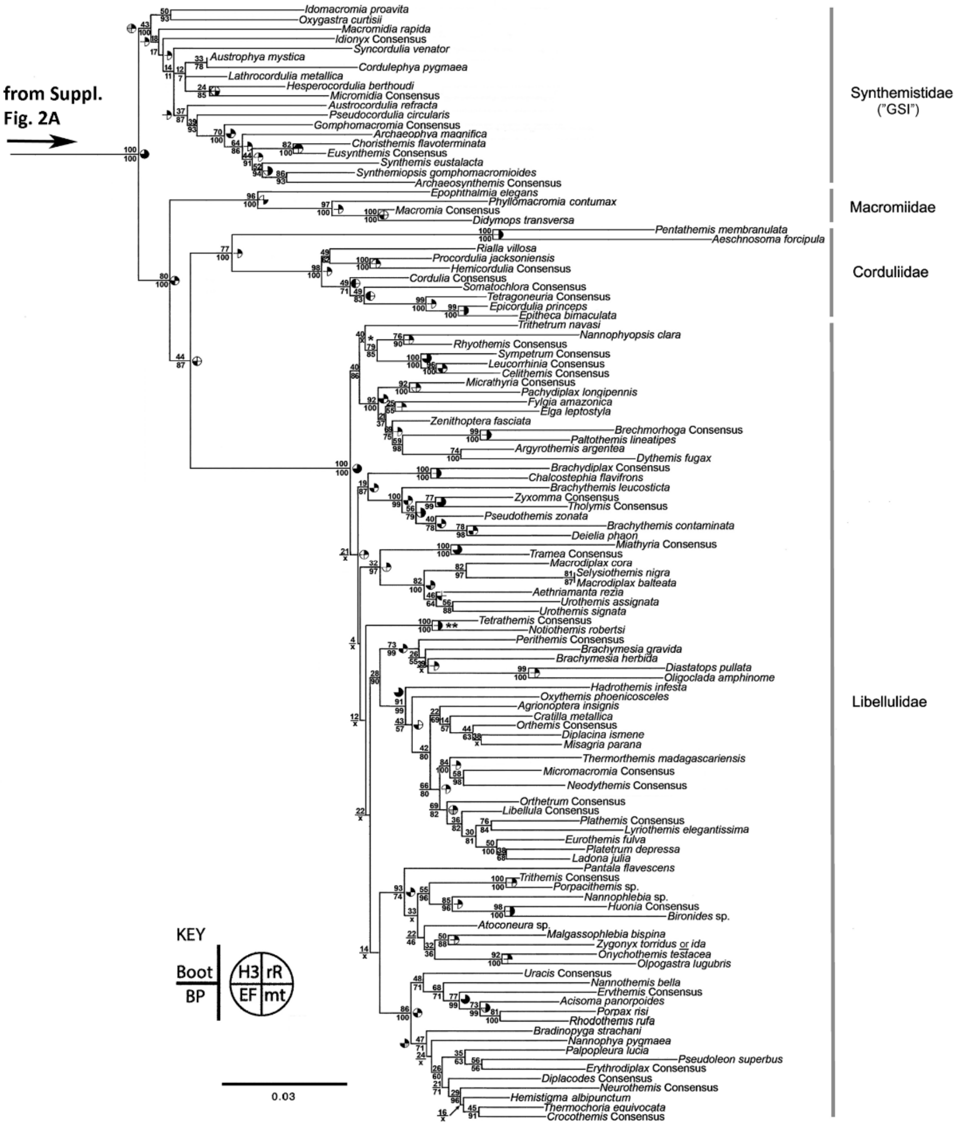

B

Figure 2. (**A**) Phylogram of non-libelluloid Anisoptera. From Carle, et al., 2015 [2]; (**B**) Phylogram of libelluloid Anisoptera. From Carle, et al. 2015 [2].

References

1. Dijkstra, K.-D.B.; Kalkman, V.J.; Dow, R.A.; Stokvis, F.R.; Van Tol, J. Redefining the damselfly families: A comprehensive molecular phylogeny of Zygoptera (Odonata). *Syst. Entomol.* **2014**, *39*, 68–96.
2. Carle, F.L.; Kjer, K.M.; May, M.L. A molecular phylogeny and classification of Anisoptera (Odonata). *Arthropod Syst. Phylogeny* **2015**, *73*, 281–301.

© 2019 by the authors. Licensee MDPI, Basel, Switzerland. This article is an open access article distributed under the terms and conditions of the Creative Commons Attribution (CC BY) license (http://creativecommons.org/licenses/by/4.0/).
